# Randomized Phase II Cancer Clinical Trials to Validate Predictive Biomarkers

**DOI:** 10.3390/biomedicines12102185

**Published:** 2024-09-26

**Authors:** Baoshan Zhang, Jong-Mu Sun, Myung-Ju Ahn, Sin-Ho Jung

**Affiliations:** 1 Department of Biostatistics and Bioinformatics, Duke University, Durham, NC 27705, USA; 2 Division of Hematology-Oncology, Department of Medicine, Samsung Medical Center, Sungkyunkwan University School of Medicine, Gangnam-gu, Seoul 135-710, Republic of Korea; jongmu.sun@samsung.com (J.-M.S.);

**Keywords:** interaction, logistic regression, sample size calculation, stratified randomization trial

## Abstract

Objectives: The design of cancer clinical trials incorporating biomarkers depends on various factors, including the trial phase, the type of biomarker and whether its role has been validated. This article aims to present a method for designing and analyzing phase II cancer clinical trials that validate predictive biomarkers. Methods: We propose a randomized trial design where patients are allocated between a targeted therapy and a non-targeted therapy stratified by biomarker status. Tumor response is used as the primary endpoint to validate the biomarker through interaction testing between treatment and biomarker positivity. Additionally we propose a sample size calculation method for this design, considering two types of interaction: one based on logit-transformed response rates and the other on raw response rates. Results: The proposed sample size method is applied to the design of a real randomized phase II trial. Extensive simulations are conducted to evaluate the performance of the test statistic and the sample size method under different scenarios. Conclusions: Our method provides a practical approach to validating predictive biomarkers in phase II cancer trials. The simulations demonstrate robust performance for both interaction models, offering guidance for the sample size selection and analysis strategy in biomarker-stratified trials.

## 1. Introduction

Cancer clinical trials often integrate a range of biomarkers that are derived from various sources including tumor tissues, blood, or urine samples. Depending on the sources, biomarkers are assessed using different methods, such as molecular, biochemical, physiological, anatomical, and imaging techniques, either at the onset of a trial or during its course. The utility of these biomarkers is multifaceted in the context of cancer diagnosis and treatment. Diagnostic biomarkers are used to diagnose cancers. For instance, carcinoembryonic antigen (CEA) is considered a diagnostic biomarker of colon cancer and rectal cancer [1]. Prognostic biomarkers are used to measure the aggressiveness of disease for patients with no or non-targeted treatment. BRAF mutation is known to be a diagnostic and prognostic biomarker for melanoma [2,3]. A predictive biomarker forecasts how well a patient might respond to a particular treatment. While BRAF mutation is a diagnostic and prognostic biomarker, it may be a predictive biomarker concerning tyrosine kinase inhibitors (TKIs), such as vemurafenib and dabrafenib, for lung cancer and melanoma patients [4].

In this paper, we focus on predictive biomarkers. Since a predictive biomarker plays a critical role in guiding therapeutic decisions for patients, it is crucial to ensure that the biomarker undergoes comprehensive validation before being employed as a treatment choice in clinical trials since an unvalidated predictive biomarker can lead to a wrong treatment for patients. In cases in which a biomarker has not yet undergone validation, it can be utilized as a stratification factor in randomized clinical trials since, by this design, the biomarker does not play any role in the selection of a treatment for patients. In such scenarios, the primary objective is to validate the biomarker rather than to use it as a basis for selecting a treatment approach. This approach ensures a more rigorous and scientifically sound application of biomarkers in clinical trial settings.

The approach to designing and analyzing clinical trials involving biomarkers can vary depending on the clinical role of the biomarker employed, its stage of development, the objectives of the study, etc. The intricate design considerations for randomized clinical trials that incorporate biomarkers were extensively explored by a group of biostatisticians [5]. Building upon this, they introduced a comprehensive set of statistical methodologies tailored for phase II randomized trials [6]. These methodologies are particularly relevant for trials involving predictive biomarkers that have yet to undergo stringent validation.

Phase II trials are pivotal and act as gatekeepers that filter out ineffective treatments before advancing to more extensive phase III studies. To be completed as quickly as possible, these trials typically select small sample sizes and a surrogate endpoint (an indicator or sign used in place of a definitive endpoint to tell if a treatment works), like tumor response or progression-free survival (PFS), rather than definitive endpoints requiring longer follow-up times, like overall survival. This approach facilitates more efficient progression through the critical phases of clinical research. In our paper, the primary endpoint is tumor response: a binary outcome.

To validate a predictive biomarker, we investigate a design method for phase II cancer clinical trials by randomizing patients between a targeted therapy and a non-targeted therapy stratified based on biomarker status (negative vs. positive). For such a trial, validation of a candidate predictive biomarker requires statistical testing of the interaction between the treatment allocation (a targeted therapy vs. a non-targeted therapy) and the biomarker status (positive vs. negative). Some investigators proposed sample size methods for case-controlled studies to test an interaction term in a logistic regression model when an exposure variable, like a biomarker, is categorical [7] and binary [8]. Their methods require inversion of a 4×4 information matrix to derive the variance of the interaction term. Using a binary predictive biomarker, we simply derive the variance using the delta method, which gives the same variance formula as the latter [8] without any matrix algebra.

In Section 2.1, we propose a sample size formula for testing the interaction between a treatment allocation and the positivity of the predictive biomarker to be validated using a logistic regression model. A logistic regression model defines an interaction in terms of the differences in logit-transformed response rates (RRs). In Section 2.2, we also present statistical testing and its sample size methods for an interaction defined by the difference between raw RRs. We demonstrate our sample size methods with the design of a real biomarker-guided trial in Section 3.1. Comprehensive numerical analyses are conducted to investigate the performance of the proposed methods in Section 3.2.

## 2. Material and Methods

Predictive biomarkers are vital for assessing the potential effectiveness of specific chemotherapy treatments. A preclinical study demonstrated that elevated levels of thymidylate synthase (TS) in tumors could indicate resistance to pemetrexed [9]. However, this hypothesis remained to be confirmed by prospective clinical research before using TS to select or deselect pemetrexed to treat patients with non-small-cell lung cancer (NSCLC). To explore this further, a phase II clinical trial was proposed to evaluate TS positivity as a predictive marker for pemetrexed/cisplatin (PC) treatment in NSCLC [10]. The trial aimed to compare the PC regimen against the standard non-targeted gemcitabine/cisplatin (GC) regimen. Participants were randomly assigned to one of the two treatment arms and were stratified based on TS status (positive vs. negative). This approach was intended to yield clearer insights into the role of TS status in predicting the efficacy of PC. The primary study endpoint was the overall response, i.e., partial response or complete response measured by RECIST [11].

### 2.1. Interaction Based on Logit-Transformed RRs

Let y=0 for non-response and 0 for response, $z_{1}=0$ for GC (non-targeted regimen) and 1 for PC (targeted regimen), and $z_{2}=0$ for TS positivity and 1 for TS negativity (the favorable biomarker status for the targeted regimen). To validate TS as a predictive biomarker for PC, we have to prove that the RR of PC depends on TS positivity, while that of GC does not. In order to test this hypothesis, we consider a logistic regression model:

(1) $$\mathrm{logit}\mathrm{Pr}\left(y=1|z_{1},z_{2}\right)=\beta_{0}+\beta_{1}z_{1}+\beta_{2}z_{2}+\beta_{3}z_{1}z_{2}$$

By model (1), the RR is $\mathrm{logit}\left(p\right)=\beta_{0}+\beta_{2}z_{2}$ for GC ($z_{1}=0$) and $\mathrm{logit}\left(p\right)=\beta_{0}+\left(\beta_{2}+\beta_{3}\right)z_{2}$ for PC ($z_{1}=1$). Since GC is not a targeted treatment, we expect its RR not to depend on the TS status ($z_{2}$), so that $\beta_{2}=0$. With $\beta_{2}=0$, $\beta_{3}$ should take a positive value if TS negativity is favorable for PC. Hence, validation of TS as a predictive biomarker of PC requires testing of $H_{0}:\beta_{3}=0$ against $H_{0}:\beta_{3}>0$.

Let $p_{kl}$ denote the RR of the patient group, with $\left(z_{1},z_{2}\right)=\left(k,l\right)$ for k,l=0 or 1. For the four combinations of $\left(z_{1},z_{2}\right)$, model (1) results in

$$\mathrm{logit}\left(p_{00}\right)=\beta_{0},\;\mathrm{logit}\left(p_{10}\right)=\beta_{0}+\beta_{1},\;\mathrm{logit}\left(p_{01}\right)=\beta_{0}+\beta_{2},\;\mathrm{logit}\left(p_{11}\right)=\beta_{0}+\beta_{1}+\beta_{2}+\beta_{3}$$

Solving these equations for $\left(\beta_{0},\beta_{1},\beta_{2},\beta_{3}\right)$ yields

(2) $$\begin{array}{cc} \beta_{0} & =\mathrm{logit}\left(p_{00}\right),\;\beta_{1}=\mathrm{logit}\left(p_{10}\right)-\mathrm{logit}\left(p_{00}\right),\;\;\beta_{2}=\mathrm{logit}\left(p_{01}\right)-\mathrm{logit}\left(p_{00}\right),\\ \beta_{3} & =\left\{\mathrm{logit}\left(p_{11}\right)-\mathrm{logit}\left(p_{10}\right)\right\}-\left\{\mathrm{logit}\left(p_{01}\right)-\mathrm{logit}\left(p_{00}\right)\right\} \end{array}$$

From Formula (2), $\beta_{3}$ is the interaction expressed in terms of logit-transformed RRs. Note that $\exp\left(\beta_{3}\right)={\mathrm{OR}}_{1}/{\mathrm{OR}}_{0}$, where ${\mathrm{OR}}_{k}=p_{k1}\left(1-p_{k0}\right)$ is the odds ratio of regimen k(=0,1) between TS positivity and TS negativity. Also, by Formula (2), we can specify regression coefficients $\left(\beta_{0},\beta_{1},\beta_{2},\beta_{3}\right)$ in terms of RRs $\left(p_{00},p_{01},p_{10},p_{11}\right)$.

We derive a test statistic for $H_{0}:\beta_{3}=0$. Let $y_{kl}$ denote the number of responders among $n_{kl}$ patients with $\left(z_{1},z_{2}\right)=\left(k,l\right)$, ${\hat{p}}_{kl}=y_{kl}/n_{kl}$, and $n=n_{00}+n_{01}+n_{10}+n_{11}$ being the total number of patients.

From Formula (2), the maximum likelihood estimator (MLE) of $\beta_{3}$ is given as

$${\hat{\beta }}_{3}=\mathrm{logit}\left({\hat{p}}_{11}\right)-\mathrm{logit}\left({\hat{p}}_{10}\right)-\mathrm{logit}\left({\hat{p}}_{01}\right)+\mathrm{logit}\left({\hat{p}}_{00}\right)$$

because ${\hat{p}}_{kl}$ is the MLE of $p_{kl}$.

Since $\sqrt{n_{kl}}\left({\hat{p}}_{kl}-p_{kl}\right)$ is approximately normal with mean 0 and variance $p_{kl}\left(1-p_{kl}\right)$, we can show that $\sqrt{n_{kl}}\left\{\mathrm{logit}\left({\hat{p}}_{kl}\right)-\mathrm{logit}\left(p_{kl}\right)\right\}$ is asymptotically normal with mean 0 and variance $1/\{p_{kl}\left(1-p_{kl}\right)\}$ using the delta method. Hence, $\sqrt{n}\left({\hat{\beta }}_{3}-\beta_{3}\right)$ is asymptotically normal with mean 0 and variance

$$n\left\{\frac{1}{n_{11}p_{11}\left(1-p_{11}\right)}+\frac{1}{n_{10}p_{10}\left(1-p_{10}\right)}+\frac{1}{n_{01}p_{01}\left(1-p_{01}\right)}+\frac{1}{n_{00}p_{00}\left(1-p_{00}\right)}\right\}$$

which converges to

(3) $$\begin{array}{c} \sigma_{3}^{2}=\left\{\frac{1}{r_{11}p_{11}\left(1-p_{11}\right)}+\frac{1}{r_{10}p_{10}\left(1-p_{10}\right)}+\frac{1}{r_{01}p_{01}\left(1-p_{01}\right)}+\frac{1}{r_{00}p_{00}\left(1-p_{00}\right)}\right\} \end{array}$$

and is consistently estimated by

(4) $$\begin{array}{c} {\hat{\sigma }}_{3}^{2}=n\left\{\frac{1}{n_{11}{\hat{p}}_{11}\left(1-{\hat{p}}_{11}\right)}+\frac{1}{n_{10}{\hat{p}}_{10}\left(1-{\hat{p}}_{10}\right)}+\frac{1}{n_{01}{\hat{p}}_{01}\left(1-{\hat{p}}_{01}\right)}+\frac{1}{n_{00}{\hat{p}}_{00}\left(1-{\hat{p}}_{00}\right)}\right\} \end{array}$$

This variance formula is identical to the (4,4)-component of the inverse of the information matrix for model (1) after complicated matrix computations by Demidenko [8]. Note that $\sigma_{3}^{2}$ decreases as the RRs, $p_{kl}$, are closer to 0.5.

Based on this result, we reject $H_{0}:\beta_{3}=0$ in favor of $H_{1}:\beta_{3}>0$ if $\sqrt{n}{\hat{\beta }}_{3}/{\hat{\sigma }}_{3}>z_{1-\alpha }$, where $z_{1-\alpha }$ is the 100(1−α) percentile of the standard normal distribution.

We want to derive a sample size formula under $H_{1}:{\bar{\beta }}_{3}\left(>0\right)$. From Formula (2), $\beta_{3}$ is expressed in terms of RRs, $p_{00},p_{01},p_{10},p_{11}$, so that ${\bar{\beta }}_{3}=\mathrm{logit}\left(p_{11}\right)-\mathrm{logit}\left(p_{10}\right)-\mathrm{logit}\left(p_{01}\right)+\mathrm{logit}\left(p_{00}\right)$ is determined once the RRs are specified. Let $a_{k}$ denote the allocation proportion to arm *k* in randomization $\left(a_{0}+a_{1}=1\right)$ and $b_{l}$ denote the prevalence of biomarker status *l* ($b_{0}+b_{1}=1$). Then, with stratified randomization, we have $r_{kl}=P\left(z_{1}=k,z_{2}=l\right)=P\left(z_{1}=k\right)P\left(z_{2}=l\right)=a_{k}b_{l}$.

For a sample size calculation, we need to specify the following input parameters.

- Type I error rate and power: (α,1−β);
- Expected RRs: $\left(p_{00},p_{01},p_{10},p_{11}\right)$;
- Allocation proportion for arm k(=0,1): $a_{k}$;
- Prevalence of biomarker status l(=0,1): $b_{l}$.

Assume that *n*, the power for $H_{1}:\beta_{3}={\bar{\beta }}_{3}\left(>0\right)$, is given by

(5) $$\begin{array}{cc} 1-\beta \; & =P\left\{\frac{\sqrt{n}{\hat{\beta }}_{3}}{{\hat{\sigma }}_{3}}>z_{1-\alpha }|H_{1}\right\}\\ \; & =P\left\{\frac{\sqrt{n}\left({\hat{\beta }}_{3}-{\bar{\beta }}_{3}\right)}{\sigma_{3}}>z_{1-\alpha }-\frac{\sqrt{n}{\bar{\beta }}_{3}}{\sigma_{3}}|H_{1}\right\} \end{array}$$

Here, the second equality holds because ${\hat{\sigma }}_{3}^{2}$ is a consistent estimator of $\sigma_{3}^{2}$.

Since $\sqrt{n}\left({\hat{\beta }}_{3}-{\bar{\beta }}_{3}\right)/\sigma_{3}$ is asymptotically N(0,1) under $H_{1}$, from Formula (5), we have

(6) $$\begin{array}{c} z_{1-\alpha }-\frac{{\bar{\beta }}_{3}\sqrt{n}}{\sigma_{3}}=-z_{1-\beta }. \end{array}$$

By solving this equation with respect to *n*, we obtain the required sample size

(7) $$n=\frac{\sigma_{3}^{2}{\left(z_{1-\alpha }+z_{1-\beta }\right)}^{2}}{{\bar{\beta }}_{3}^{2}}$$

Recall that by Formula (3), $\sigma_{3}^{2}$ is expressed in terms of $\left(p_{00},p_{01},p_{10},p_{11}\right)$, $a_{0}$, and $b_{0}$.

### 2.2. Interaction Based on Raw RRs

We considered an interaction in terms of logit-transformed RRs in Section 2.1. In this section, we consider an interaction in terms of raw RRs, $\theta =\left(p_{11}-p_{10}\right)-\left(p_{01}-p_{00}\right)$. We have θ=0 if the biomarker is not a predictive biomarker for regimen $z_{1}=1$. So we derive a test statistic and its sample size formula for testing $H_{0}:\theta =0$.

The interaction θ is estimated by

$$\hat{\theta }={\hat{p}}_{11}-{\hat{p}}_{10}-{\hat{p}}_{01}+{\hat{p}}_{00}$$

By the binomial theory, $\sqrt{n_{kl}}\left({\hat{p}}_{kl}-p_{kl}\right)$ is approximately normal with mean 0 and variance $p_{kl}\left(1-p_{kl}\right)$ for large values of *n*; so for $H_{0}:\theta =0$, $\sqrt{n}\hat{\theta }$ is asymptotically normal with mean 0 and variance

$$\sigma_{n}^{2}=n\left\{\frac{p_{11}\left(1-p_{11}\right)}{n_{11}}+\frac{p_{10}\left(1-p_{10}\right)}{n_{10}}+\frac{p_{01}\left(1-p_{01}\right)}{n_{01}}+\frac{p_{00}\left(1-p_{00}\right)}{n_{00}}\right\}$$

which is consistently estimated by

$${\hat{\sigma }}^{2}=n\left\{\frac{{\hat{p}}_{11}\left(1-{\hat{p}}_{11}\right)}{n_{11}}+\frac{{\hat{p}}_{10}\left(1-{\hat{p}}_{10}\right)}{n_{10}}+\frac{{\hat{p}}_{01}\left(1-{\hat{p}}_{01}\right)}{n_{01}}+\frac{{\hat{p}}_{00}\left(1-{\hat{p}}_{00}\right)}{n_{00}}\right\}$$

Note that θ>0 if the biomarker is predictive for regimen $z_{1}=1$. So we reject $H_{0}:\theta =0$ in favor of $H_{1}:\theta >0$ if $\sqrt{n}\hat{\theta }/\hat{\sigma }>z_{1-\alpha }$. Similarly to Section 2.1, we can derive the required sample size for $H_{1}:\theta =\bar{\theta }\left(>0\right)$

(8) $$\begin{array}{c} n=\frac{\sigma^{2}{\left(z_{1-\alpha }+z_{1-\beta }\right)}^{2}}{{\bar{\theta }}^{2}} \end{array}$$

where

$$\sigma^{2}=\frac{p_{11}\left(1-p_{11}\right)}{r_{11}}+\frac{p_{10}\left(1-p_{10}\right)}{r_{10}}+\frac{p_{01}\left(1-p_{01}\right)}{r_{01}}+\frac{p_{00}\left(1-p_{00}\right)}{r_{00}}$$

is the limit of $\sigma_{n}^{2}$. Unlike the variance of ${\hat{\beta }}_{3}$, $\sigma^{2}$ increases as the RRs, $p_{kl}$, are closer to 0.5.

## 3. Numerical Analysis

### 3.1. Real Study Example

We apply our sample size calculation methods to the design of the randomized phase II trial between GC and PC stratified by TS status (positive vs. negative) [10]. Patients are randomized between the two treatment arms in a one-to-one ratio, i.e., $a_{0}=a_{1}=1/2$, and are stratified by TS status to make sure that the two treatment arms are balanced within both the TS positive and TS negative cohorts. The cutoff value for TS positivity is selected as the median value from a previous study by Sun et al. [12], so we expect $b_{0}=b_{1}=1/2$ and $r_{kl}=a_{k}b_{l}=1/4$ for k,l=0,1. From this previous study, the investigators observed ${\hat{p}}_{00}=0.37$, ${\hat{p}}_{10}=0.24$, ${\hat{p}}_{01}=0.32$, and ${\hat{p}}_{11}=0.48$. The fact that ${\hat{p}}_{00}\approx {\hat{p}}_{01}$ implies that GC ($z_{1}=0$) is a non-targeted treatment, whereas ${\hat{p}}_{11}>{\hat{p}}_{10}$ implies that TS negativity $\left(z_{2}=1\right)$ is favorable for PC $\left(z_{1}=1\right)$. We use these estimates as the true RRs specified for our sample size calculation to test if TS is a predictive biomarker for PC or not.

Using the interaction defined in terms of logit-transformed RRs, we calculate the sample size for $H_{1}:\beta_{3}={\bar{\beta }}_{3}\left(>0\right)$. Based on the specified RRs, we have ${\bar{\beta }}_{3}=1.294$ from Formula (2). Additionally, by incorporating $r_{kl}=1/4$, we obtain $\sigma_{3}^{2}=73.50$ from Formula (3). Hence, for (α,1−β)=(0.1,0.9), the required sample size is

$$n=\frac{73.50\times {\left(1.282+1.282\right)}^{2}}{1.294^{2}}=289$$

from Formula (7).

Now, we calculate the sample size for testing $H_{0}:\theta =0$ using the interaction based on the differences among raw RRs. For the specified RRs and $r_{kl}=1/4$, we have $\bar{\theta }=0.290$ and $\sigma^{2}=3.531$. Hence, for (α,1−β)=(0.1,0.9), the required sample size is

$$n=\frac{3.531\times {\left(1.282+1.282\right)}^{2}}{0.290^{2}}=276$$

### 3.2. Simulations

In this subsection, we conduct extensive simulations to show that the statistical test of the interaction term accurately controls the type I error rate and that a calculated sample size is appropriately powered.

At first, the sample size *n* is calculated for a given design setting under $H_{1}$ and for 10,000 simulation samples of size *n* under the design setting. Statistical testing is applied to each sample to calculate the empirical power $1-\hat{\beta }$ by the proportion of samples rejecting $H_{0}$ among the 10,000 simulation samples. We will conclude that our sample size formula is accurate if the empirical power is close to the nominal one. We also generate 10,000 samples of size *n* under $H_{0}$ and calculate the empirical type I error rate $\hat{\alpha }$ similarly. If the empirical type I error rate $\hat{\alpha }$ is close to the nominal α, we will conclude that the test statistic controls the type I error rate accurately for the calculated sample size.

**Figure 1:**
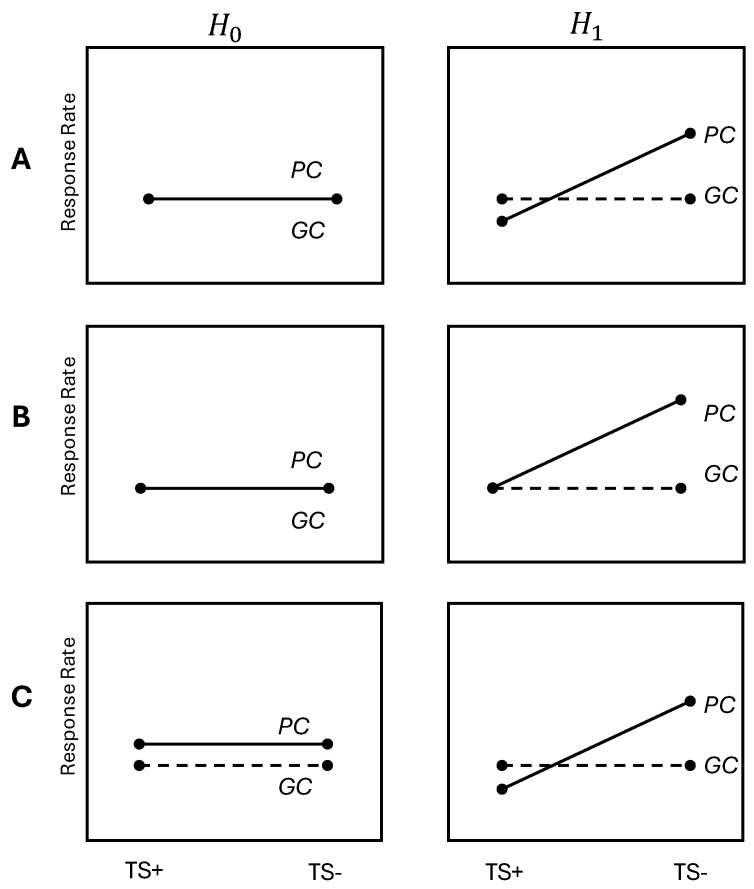
Three scenarios (**A**–**C**) of RRs for GC and PC depending on TS status under $H_{0}$ and $H_{1}$.

We set (α,1−β)=(0.1,0.9) and $a_{00}=a_{01}=a_{10}=a_{11}=1/4$. In addition, we consider three different scenarios (A, B, and C) for $H_{0}$ and $H_{1}$. In Table 1, four sets of $\left(p_{00},p_{01},p_{10},p_{11}\right)$ values are given for $H_{0}$ and $H_{1}$ for each scenario. Figure 1 displays a typical set of RRs for each scenario. Since the line of GC (connecting $p_{00}$ and $p_{01}$) is horizontal under both $H_{0}$ and $H_{1}$, it is a non-targeted treatment. The line of PC (connecting $p_{10}$ and $p_{11}$) is also horizontal under $H_{0}$ but not under $H_{1}$, so we want to test if PC is a targeted treatment or not. In scenarios A and B under $H_{0}$, we assume that the relative risk (RR) values for GC and PC are equal, and in scenario C, we assume both lines are horizontal but have different values. Under $H_{1}$, in scenarios A and C, we assume the RR value of PC is lower than that of GC for TS+ patients but higher for TS− patients. In scenario B under $H_{1}$, we assume that PC and GC have the same RR value for TS+ patients but that PC has a higher RR value than GC for TS− patients. More details about the parameter settings for the simulations can be found in Table 1.

Table 2 summarizes the odds ratios under $H_{1}$, the sample sizes, and the simulation results under these design settings. Note that sample size decreases based on the size of the interaction for both types of interaction. If the interaction under $H_{1}$ is identical, the sample size decreases (increases) if the RRs are closer to 0.5 for θ (for θ) because of the relationship between the variance of the interaction estimator and the RRs. We observe that for each design scenario, the sample size for testing $H_{0}:\theta =0$ is smaller than that for testing $H_{0}:\theta =0$, probably because the RRs under each $H_{1}$ exactly satisfy the interaction based on the raw RRs. For all of the $H_{1}$ scenarios, the empirical power $1-\hat{\beta }$ is close to the nominal 1−β=0.9, so we conclude that our sample size formulas are accurate. The empirical type I error rates $\hat{\alpha }$ are close to the nominal α=0.1, so we conclude that the test statistics control the type I error rate accurately for the wide range of sample sizes we have considered.

## 4. Discussion

A biomarker cannot be used to select a treatment until it is validated because it is very risky to select a patient’s treatment based on a wrong biomarker. However, a biomarker can be used as a stratification factor for a randomization trial even before validated. We investigated design and analysis methods for a randomized trial stratifying for biomarker positivity. This trial tests whether or not the biomarker is a predictive biomarker for a treatment by using a non-targeted treatment as a control. The analysis of the trial requires statistical testing on the interaction between treatment allocation and biomarker positivity.

We derived statistical tests and their sample size methods for two different forms of interactions: one based on the logit-transformed RRs and the other based on the raw RRs of subgroups defined by $\left(z_{1},z_{2}\right)$. Although we presented our methods for phase II trials, they can be used for phase III trials, too.

Extensive simulations were conducted under scenarios of alternative hypotheses for which the required sample sizes were reasonable for a real phase II trial to validate a potential predictive biomarker. The sample sizes calculated using our formulas were found to be appropriately powered. Also, from simulations under $H_{0}$, our test statistic controlled the type I error rate accurately for the range of sample sizes calculated.

In our paper, we wanted to test if a treatment is a targeted therapy for a biomarker or not. A simple design for this objective might be a single-arm design using a treatment group and a test group if the RR of the treatment is different between the biomarker positive group and the biomarker negative group. Instead, we selected a randomized trial design between a candidate targeted therapy arm and a non-targeted therapy arm (control) stratified by the status of the biomarker. By this design, the required sample size will increase, but as a secondary objective, we tested if the selected control treatment was really a non-targeted therapy with respect to the biomarker or not. This can be done by testing $\beta_{2}$ in model (1). Furthermore, by the stratification based on the biomarker status, the biomarker outcome was not used for the selection of the treatment for each patient. If the biomarker is shown to be a predictive biomarker for a treatment, then we can consider proceeding to a randomized phase III trial between a biomarker-guided treatment selection arm as an experimental therapy and a control arm using a treatment selected at the discretion of the treating physician and blinded in terms of biomarker status. If the biomarker is proven to be predictive for the targeted therapy through these trials, patients with a biomarker status that is beneficial in terms of the therapy will be treated with this therapy, resulting in personalized medicine for the target population.

Our computer programs for sample size calculation and simulations were developed in R and are available upon request.

## Figures and Tables

**Table 1:** Hypothesis settings under different scenarios.

	$H_{0}$				$H_{1}$			
Scenario	$p_{00}$	$p_{01}$	$p_{10}$	$p_{11}$	$p_{00}$	$p_{01}$	$p_{10}$	$p_{11}$
A1	0.2	0.2	0.2	0.2	0.2	0.2	0.1	0.4
A2	0.3	0.3	0.3	0.3	0.3	0.3	0.2	0.5
A3	0.4	0.4	0.4	0.4	0.4	0.4	0.3	0.6
A4	0.5	0.5	0.5	0.5	0.5	0.5	0.4	0.7
B1	0.2	0.2	0.2	0.2	0.2	0.2	0.2	0.55
B2	0.3	0.3	0.3	0.3	0.3	0.3	0.3	0.65
B3	0.4	0.4	0.4	0.4	0.4	0.4	0.4	0.75
B4	0.5	0.5	0.5	0.5	0.5	0.5	0.5	0.85
C1	0.2	0.2	0.3	0.3	0.2	0.2	0.1	0.5
C2	0.3	0.3	0.4	0.4	0.3	0.3	0.2	0.6
C3	0.4	0.4	0.5	0.5	0.4	0.4	0.3	0.7
C4	0.5	0.5	0.6	0.6	0.5	0.5	0.4	0.8

**Table 2:** Interactions under $H_{1}$, sample size, empirical type I error rate $\hat{\alpha }$, and power $1-\hat{\beta }$ under different design settings.

	$H_{0}:\beta_{3}=0$				$H_{0}:\theta =0$			
Scenario	${\bar{\beta }}_{3}$	n	$\hat{\alpha }$	$1-\hat{\beta }$	$\bar{\theta }$	n	$\hat{\alpha }$	$1-\hat{\beta }$
A1	1.792	228	0.1005	0.9180	0.30	188	0.1048	0.9033
A2	1.386	272	0.1013	0.9028	0.30	244	0.1038	0.8932
A3	1.253	288	0.1043	0.9086	0.30	272	0.0984	0.8983
A4	1.253	284	0.1077	0.9074	0.30	276	0.1030	0.8958
B1	1.587	236	0.1023	0.9004	0.35	156	0.1091	0.8949
B2	1.466	228	0.1004	0.9095	0.35	184	0.1025	0.8986
B3	1.504	208	0.0974	0.9094	0.35	196	0.1092	0.9001
B4	1.735	172	0.0979	0.9142	0.35	188	0.1003	0.8974
C1	1.792	152	0.1011	0.8962	0.30	108	0.1085	0.8912
C2	2.197	164	0.0997	0.9057	0.40	136	0.1088	0.902
C3	1.792	164	0.096	0.9056	0.40	148	0.1083	0.8972
C4	1.792	152	0.108	0.9143	0.40	148	0.1019	0.8969

## Data Availability

Not applicable.

## Abbreviations

The following abbreviations are used in this manuscript:

TS	Thymidylate Synthase
PC	Pemetrexed/Cisplatin
GC	Gemcitabin/Cisplatin
NSCLC	Non-small-cell Lung Cancer
RR	Response Rate
